# Comparing the National Early Warning Score and the Manchester Triage System in Emergency Department Triage: A Multi-Outcome Performance Evaluation

**DOI:** 10.3390/diagnostics15091055

**Published:** 2025-04-22

**Authors:** Arian Zaboli, Serena Sibilio, Gloria Brigiari, Magdalena Massar, Norbert Pfeifer, Francesco Brigo, Gianni Turcato

**Affiliations:** 1 Innovation, Research and Teaching Service (SABES-ASDAA), Teaching Hospital of the Paracelsus Medical Private University (PMU), 39100 Bolzano, Italy; magdalena.massar@sabes.it (M.M.); francesco.brigo@sabes.it (F.B.); 2 Department Public Health, Institute of Nursing Science, Universität Basel, 4051 Basel, Switzerland; serena.sibilio@unibas.ch; 3 Unit of Biostatistics, Epidemiology and Public Health, Department of Cardiac, Thoracic, Vascular Sciences and Public Health, University of Padova, 35122 Padova, Italy; gloria.brigiari@ubep.unipd.it; 4 Department of Emergency Medicine, Hospital of Merano-Meran (SABES-ASDAA), 39012 Merano-Meran, Italy; norbert.pfeifer@sabes.it; 5 Intermediate Care Unit, Department of Internal Medicine, Hospital Alto Vicentino (AULSS-7), 36014 Santorso, Italy; gianni.turcato@yahoo.it; 6 Department of Health Sciences, UniCamillus-Saint Camillus International University of Health Science, 00131 Rome, Italy

**Keywords:** triage, early warning score, Manchester triage system, risk assessment, patient prioritization, clinical decision-making

## Abstract

**Background:** Emergency department (ED) triage systems aim to prioritize patients based on clinical severity, ensuring timely intervention for high-risk cases. Recently, the National Early Warning Score (NEWS) has been proposed as an alternative to traditional triage systems, but its efficacy across multiple clinical outcomes remains unclear. This study aimed to compare the predictive performance of the NEWS and the Manchester Triage System (MTS) across multiple clinical outcomes. **Methods:** We conducted a retrospective, single-center study at Merano Hospital, Italy, from 1 June 2022 to 30 June 2023, comparing the performance of the NEWS and the Manchester Triage System (MTS). All adult ED patients (≥18 years) were included, while exclusions applied to those on fast-track pathways, non-residents, and pregnant patients. Primary outcomes included 30-day mortality, hospitalization, and ICU admission. A random 5% subgroup was analyzed for secondary outcomes, including the need for life-saving interventions (LSIs), physician-defined clinical priority, and severity. Predictive performance was assessed using Receiver Operating Characteristic (ROC) curves, area under the ROC curve (AUROC) comparisons, and Decision Curve Analysis (DCA). **Results:** Among 27,238 patients, the NEWS predicted 30-day mortality more accurately than the MTS (AUROC 0.745 vs. 0.701, *p* < 0.001). However, the MTS outperformed the NEWS for hospitalization (AUROC 0.733 vs. 0.609, *p* < 0.001), ICU admission (AUROC 0.862 vs. 0.672, *p* < 0.001), and all secondary outcomes. DCA further confirmed MTS’s superiority across clinically relevant ED probability thresholds (20–40%). **Conclusions:** The NEWS, while effective for predicting mortality, it is inadequate in comprehensive triage decision-making. The MTS remains the superior system for prioritizing high-risk patients based on clinical severity. Rather than replacing triage with the NEWS, efforts should focus on refining existing systems to improve risk stratification. Future multi-center prospective studies are necessary to validate these findings.

## 1. Introduction

Triage in the emergency department (ED) is essential for identifying life-threatening conditions and ensuring that high-risk patients receive immediate care, while those who can safely wait are appropriately managed [1]. Despite this critical function, current triage systems are often criticized for their limited ability to accurately assess patient severity [2,3,4].

Some of these criticisms are justified, as the most widely used triage systems undergo minimal substantial updates [2,4]. As a result, they fail to improve over time, despite an increasing body of scientific evidence [2,4,5,6]. Additionally, recent literature reviews have shown that, among validated and extensively studied triage systems, no significant differences exist in classification accuracy [5,6]. Consequently, efforts have been made to enhance existing triage methods by integrating complementary tools to improve predictive capabilities [7,8].

One of the most debated approaches is the incorporation of the National Early Warning Score (NEWS) for all patients at triage, aiming to improve severity assessment through the systematic evaluation of vital signs [9,10]. Currently, no triage system mandates the collection of all vital signs for every patient [5,6]. This is primarily due to the heterogeneous nature of the ED population: requiring such measurements for all cases may not always be appropriate and could unnecessarily prolong triage times, potentially compromising its effectiveness.

Nevertheless, recent studies comparing the NEWS with traditional triage systems have supported its use, suggesting that it could replace conventional triage [9,11]. However, available studies primarily rely on outcomes such as 30-day mortality and hospitalization rates [9,11]. While these indicators are relevant, 30-day mortality does not always have a direct causal relationship with triage coding, and hospitalization decisions are influenced by subjective factors as well as hospital-specific structural and organizational characteristics.

This study was specifically conducted with the aim of evaluating whether the NEWS could serve as a reliable alternative to a traditional triage system, specifically the Manchester Triage System (MTS), by comparing their accuracy across multiple clinically relevant outcomes.

## 2. Methods

### 2.1. Study Design and Setting

This retrospective, single-center study was conducted from 1 June 2022 to 30 June 2023, at the ED of Merano Hospital, Italy.

In this ED, all patients undergo a triage evaluation using the Manchester Triage System (MTS), one of the most extensively studied and validated triage systems. Implemented since 2014, the MTS assigns patients one of five priority levels based on their presenting complaint, which is categorized within a decision-making diagram that includes specific indicators. The presence of at least one positive indicator determines the assigned priority code, which corresponds to a maximum waiting: from 240 min for priority code 5 to immediate cares (0 min) for priority code 1.

All triage nurses working in our ED have at least two years of critical care experience and have completed a two-day specialized MTS training course, followed by supervised training with an experienced triage nurse. Currently, 26 triage-certified nurses actively perform triage assessments in our ED.

### 2.2. Participants

This study utilized data from an internal quality improvement project conducted at Merano Hospital. For all adult patients (≥18 years old) who underwent triage during the study period were considered eligible. For each patient, the triage nurse systematically recorded all vital signs required for calculating the NEWS [12], while also assigning a priority code according to the MTS.

Therefore, each patient in the study population was classified using both the NEWS and MTS, allowing for a direct, within-subject comparison of the two systems without the need for separate study groups. Importantly, patient management and clinical pathways were based solely on the MTS-assigned priority code, as the NEWS was recorded for research and quality improvement purposes only, without influencing triage decisions or care delivery.

The study dataset was extracted from an electronic database developed for the quality improvement project, applying the following exclusion criteria: (a) patients referred via fast-track pathways, meaning those directly sent from triage to a specialty outpatient clinic. This was necessary as their care pathway differed from standard ED management, and they exited the ED system; (b) non-residents of the study district, as their clinical outcomes could not be reliably tracked; (c) pregnant or suspected pregnant patients; (d) cases with incomplete or incorrect vital sign data.

All eligible patients were consecutively enrolled throughout the study period, and no formal sample size calculation was performed.

### 2.3. Quality Improvement Protocol

During the study period, the ED implemented a quality improvement initiative requiring systematic NEWS calculation for all patients at triage. The only exclusion criterion was patients in critical condition who were immediately transferred to the resuscitation room with full emergency team activation.

For all other patients, in addition to standard triage assessment, the following vital parameters were collected and recorded for NEWS calculation: (1) respiratory rate; (2) oxygen saturation; (3) body temperature; (4) systolic blood pressure; (5) heart rate; (6) consciousness level, assessed using the AVPU scale; (7) fraction of inspired oxygen (FiO_2_).

To facilitate data collection, the electronic nursing assessment form was updated to integrate the NEWS calculation within the standard triage workflow. The collected data were recorded within the triage electronic records and extracted using QlikView 12.40 (QlikTech) management software. All data were extracted from the hospital’s integrated electronic medical records system using QlikView (QlikTech), a business intelligence software adopted by the ED of the Merano Hospital for clinical and administrative data management. This system collects and stores patient information in real time, including triage assessments, clinical notes, vital signs, and outcome data.

### 2.4. Variables and Subgroup Analysis

From the electronic database, all enrolled patients were extracted, and the following variables were collected for each case: (1) NEWS; (2) age; (3) mode of ED arrival; (4) time of arrival; (5) triage priority code assigned; (6) need for general ward hospitalization; (7) need for ICU admission; (8) 30-day mortality.

Given the large patient volume, a full manual reconstruction of detailed clinical histories for all cases was not feasible. Therefore, a subgroup analysis was conducted on a randomly selected 5% sample of the overall study population. The sample was extracted using STATA 16.1, assigning a random number to each patient and applying a fixed random seed to ensure reproducibility. The extracted sample maintained the distribution of triage urgency levels to ensure representativeness.

For this subset of patients, additional clinical data were collected through detailed manual chart review, performed by two emergency physicians and two triage-trained nurses.

Nurses reconstructed the Charlson Comorbidity Index (CCI) and Acute Physiologic Assessment and Chronic Health Evaluation (APACHE) score and identified cases requiring life-saving interventions (LSI), as defined by Platts-Mills et al. [13].

Physicians assessed two key outcomes: clinical priority at admission, evaluating whether the patient truly required ED care (binary outcome) [14]; clinical severity, considering the full diagnostic and therapeutic pathway to determine whether the patient’s condition justified ED management (binary outcome) [14].

### 2.5. Outcomes

Primary outcomes (analyzed for the entire study population):

30-day mortality: all-cause death within 30 days of ED admission, reconstructed using hospital registry data [14].

Hospitalization: need for general ward admission following ED evaluation [5,6,14].

ICU admission: need for ICU admission after ED assessment [5,6,14].

Secondary outcomes (analyzed in the 5% random subgroup):

Life-saving intervention (LSI)*:* defined as (1) airway and breathing support (intubation or emergent noninvasive positive pressure ventilation); (2) electrical therapy (defibrillation, emergent cardioversion, or external pacing); (3) procedures (chest needle decompression, pericardiocentesis, or open thoracotomy); (4) hemodynamic support (significant intravenous fluid resuscitation in the setting of hypotension, blood administration, or control of major bleeding); (5) emergency medications (naloxone, dextrose, atropine, adenosine, epinephrine, or vasopressors) [13]. LSIs were reconstructed through manual chart review by nurses.

Clinical priority: presence of symptoms truly requiring ED evaluation, reconstructed by ED physicians based on triage records and initial assessments [14].

Clinical severity: evaluated by ED physicians through a comprehensive review of all clinical records and diagnostic tests performed during ED stay [14].

### 2.6. Ethical Considerations

This study was approved by the Ethics Committee for Clinical Research, South Tyrol Health Authority, Bolzano, Italy (Approval number: 28-2024) and was conducted in accordance with the Declaration of Helsinki principles for ethical medical research involving human subjects. As this study was based on an internal clinical improvement project, patients were not informed about their participation. Thus, no formal consent was required.

### 2.7. Statistical Analysis

The distribution of continuous variables was visually assessed using histograms, given the large dataset. Continuous variables were reported as mean ± standard deviation (SD) or median with interquartile range (IQR), depending on data distribution. Univariate comparisons were conducted using the Mann–Whitney test for non-parametric data. Categorical variables were expressed as absolute numbers and percentages and compared using Fisher’s exact test or the chi-square test, as appropriate.

To compare the classification performed by the MTS and NEWS, both variables were categorized into three groups.

The NEWS was categorized as low-score (NEWS 0–4), medium-score (NEWS 5–6), and high-score (NEWS ≥ 7) [12].

The MTS was categorized as high-priority (codes 1–2), medium-priority (code 3), and low-priority (codes 4–5).

The classifications of the MTS and NEWS were compared using a 3 × 3 contingency table across the general study population.

The predictive performance of the NEWS and MTS was assessed using Receiver Operating Characteristic (ROC) curves, and the area under the ROC curve (AUROC) comparisons for each outcome were performed using DeLong’s test.

Finally, Decision Curve Analysis (DCA) was performed for all study outcomes [15]. DCA evaluates the clinical benefit of predictive models and optimizes clinical decision-making strategies [15]. Based on DCA, the net clinical benefit of using the NEWS or MTS in triage was assessed over a range of threshold probabilities for patient risk stratification. Due to the low incidence of ICU admissions and LSI, DCA was not performed for these outcomes.

Statistical tests with *p*-values < 0.05 were considered significant. All analyses were conducted using STATA 16.1 (StataCorp LLC, College Station, TX, USA. 2019. Stata Statistical Software: Release 16.1).

## 3. Results

**Figure 1:**
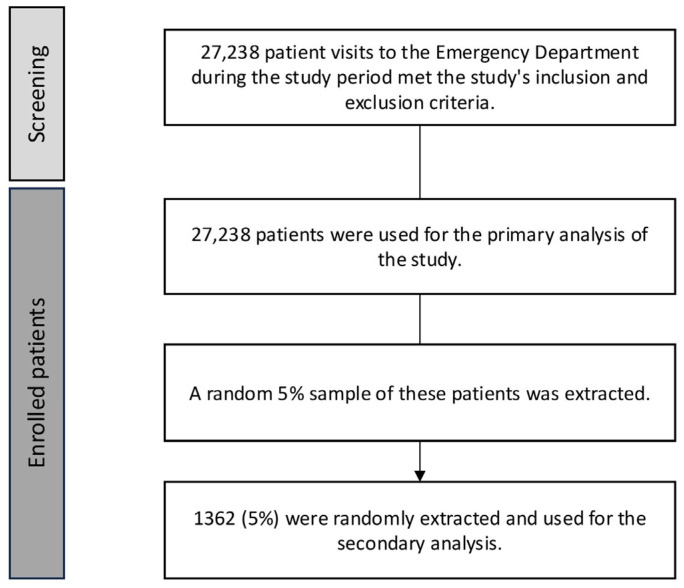
Flow chart of patient enrollment and selection process for primary and secondary analyses.

During the study period, 27,238 patients were enrolled (Figure 1). Among them, 91.3% (24,866/27,238) had a NEWS between 0 and 4, 5.2% (1417/27,238) had a NEWS between 5 and 6, and 3.5% (955/27,238) had a NEWS ≥ 7. The characteristics of the enrolled patients are summarized in Table 1.

Patients with higher NEWSs were more frequently transported to the ED by ambulance, whereas those with lower NEWSs predominantly arrived independently (Table 1). A similar trend was observed for triage codes: higher NEWSs correlated with more severe triage codes, whereas lower NEWSs were associated with less severe codes (Table 1).

Moreover, as the NEWS increased, the incidence of all three outcomes (hospital admission, ICU admission, and 30-day mortality) increased significantly (Table 1).

Table S1 presents a contingency table comparing NEWS categories with triage codes. Among patients with low NEWSs (0–4), 70.7% were assigned a low-priority triage code (4–5). However, 6.2% received a high-priority triage code despite a low NEWS. Conversely, among patients with high NEWSs (≥7), 67.1% received a high-priority triage code, while 5.4% were assigned a low-priority triage code despite their high NEWS (Table S1).

The predictive ability of the NEWS and triage was evaluated using ROC curves for study outcomes (Table 2). For 30-day mortality, the NEWS demonstrated superior predictive accuracy compared to the triage system (0.745 vs. 0.701, *p* < 0.001). However, for hospitalization and ICU admission, the triage system significantly outperformed the NEWS, with AUROC values of 0.733 vs. 0.609 (*p* < 0.001) for hospitalization and 0.862 vs. 0.672 (*p* < 0.001) for ICU admission.

**Figure 2:**
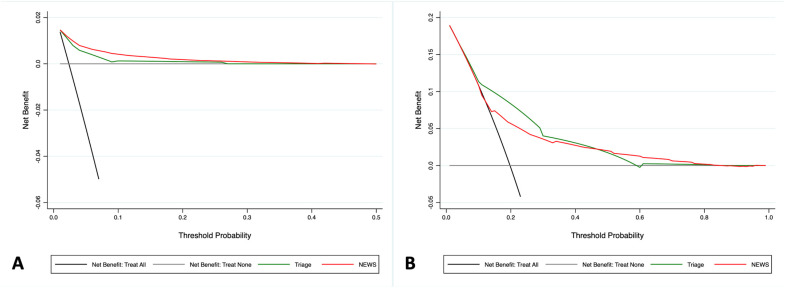
Decision Curve Analysis comparing the net clinical benefit of triage (green line) and the NEWS (red line) in ED patients. The x-axis represents the threshold probability for adverse cardiac events, while the y-axis indicates the net benefit. The black line assumes that all the patients would have the outcome, whereas the gray line assumes that no patients will experience the outcome. The green line represents the net clinical benefit provided by the triage evaluation, and the red line represents the net clinical benefit provided by the NEWS. In panel (**A**), the NEWS demonstrates greater clinical utility for predicting 30-day mortality, whereas in panel (**B**), triage outperforms the NEWS in predicting hospitalization within clinically relevant probability thresholds.

The DCA for mortality and hospital admission is presented in Figure 2. For 30-day mortality, the NEWS demonstrated a greater net clinical benefit compared to the triage system. In contrast, for hospital admission, the MTS outperformed the NEWS, particularly at clinically relevant probability thresholds between 20% and 40%. At higher thresholds, the NEWS showed a slight advantage, but these are less applicable in routine ED practice (Figure 2).

A randomly selected 5% sample (*n* = 1362) of the total study population was analyzed. Their characteristics are reported in Table S2, and their distribution was comparable to the overall cohort in terms of demographic and clinical parameters. Patients with higher NEWSs had significantly higher Charlson Comorbidity Index (CCI) values, indicating a greater burden of comorbidities (Table S2). A similar trend was found for the APACHE score, which increased with more severe vital sign abnormalities. Additionally, all study outcomes in this subgroup were significantly associated with higher NEWSs (Table S2).

The ROC curves for the secondary outcomes in the subgroup analysis are reported in Table 3. The triage system demonstrated superior predictive performance compared to the NEWS across all secondary outcomes. For LSI, the MTS had a significantly higher AUROC compared to the NEWS (0.835 vs. 0.751, *p* < 0.001). For physician-defined urgency, the MTS outperformed the NEWS (AUROC: 0.857 vs. 0.656, *p* < 0.001), whereas for physician-defined severity, the MTS also demonstrated better accuracy (AUROC: 0.767 vs. 0.661, *p* < 0.001).

**Figure 3:**
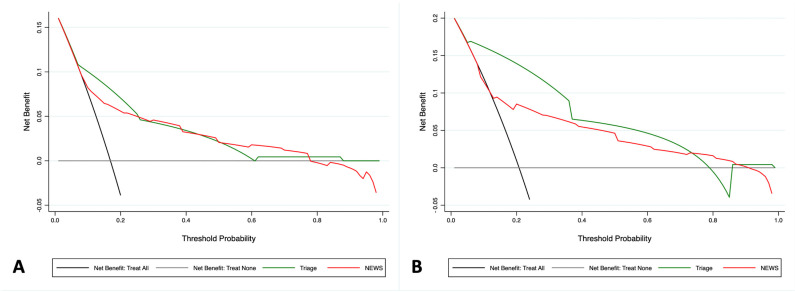
Decision Curve Analysis comparing the net clinical benefit of triage (green line) and the NEWS (red line) in ED patients. The x-axis represents the threshold probability for adverse cardiac events, while the y-axis indicates the net benefit. The black line assumes that all patients will experience the outcome, whereas the gray line assumes that no patients will experience the outcome. The green line represents the net clinical benefit provided by the triage evaluation, and the red line represents the net clinical benefit of the NEWS. As shown in panels (**A**,**B**), triage demonstrated greater clinical utility in predicting both clinical priority and clinical severity within relevant threshold probability ranges.

Finally, DCA was performed for clinical urgency and clinical severity in this subgroup (Figure 3). Again, the MTS demonstrated superior performance compared to the NEWS, particularly within probability thresholds relevant to the emergency department (20–40%). In both cases, the MTS maintained a net clinical benefit greater than 5% compared to the NEWS at a probability threshold of approximately 20% (Figure 3).

## 4. Discussion

This study is the first to compare the performance of a triage system like the MTS with an early warning score such as the NEWS, considering multiple outcomes beyond just 30-day mortality. Our findings demonstrate that the MTS stratifies risk significantly better than the NEWS across all study outcomes, except for 30-day mortality. This conclusion remains valid despite reducing the standard five-level triage classification to three levels to facilitate comparison with the NEWS. Unlike previous studies, our results do not support replacing triage systems with early warning scores like the NEWS [9,11].

Our study has several important implications for clinical practice. First, with the exception of 30-day mortality, the NEWS consistently performed worse than the MTS across all outcomes. This is likely because triage prioritization reflects the clinical and symptomatic severity of the patient at initial assessment, whereas the NEWS was designed to identify patients at risk of clinical deterioration and short-term mortality [12,16]. However, symptom severity does not necessarily predict negative clinical outcomes [16].

The choice of the most appropriate outcome for evaluating triage systems has long been debated [14,16,17]. On one hand, 30-day mortality is influenced by numerous post-triage factors, while other clinically relevant outcomes, such as the patient’s clinical severity, are often difficult to reconstruct [16,17]. For this reason, many previous studies have focused on a single outcome while neglecting others. For instance, Schinkel et al. compared the NEWS with the Netherlands’ national triage system using only 30-day mortality and hospitalization rates [11]. Their findings suggested that the NEWS outperformed the Dutch triage system in these aspects, but the study did not assess more appropriate indicators of clinical severity, leading to criticism regarding its limited scope [11]. As suggested by Kuriyama et al., a comprehensive evaluation of triage systems requires multiple clinical outcomes rather than relying on a single endpoint [17].

In our study, the NEWS outperformed the MTS only for 30-day mortality, which is expected since the NEWS was explicitly designed to identify patients at risk of death [11]. Conversely, triage systems were developed to prioritize patients with symptoms indicative of severe, time-sensitive conditions [14,16]. Moreover, in the ED setting, mortality has a relatively low prevalence [14].

A large observational study from northern Denmark reported that 30-day ED mortality is approximately 3.0% (95% CI: 2.9–3.1), while 1-day mortality is around 0.5% (95% CI: 0.5–0.5) [18]. This low mortality rate is well recognized, even in resource-limited settings [19]. For example, a two-year study in Ethiopia found an ED mortality rate of 2.8%, with 71.4% of deaths occurring within 72 h of arrival [19].

Given this consistently low incidence, multiple studies have highlighted that mortality is not the most appropriate outcome for evaluating triage systems [14]. Consequently, studies focusing primarily on mortality as their primary endpoint provide limited insights into triage systems effectiveness. A more comprehensive evaluation should include additional clinically relevant outcomes to assess a triage system’s ability to prioritize critically ill patients.

Our subgroup analyses confirm that the MTS significantly outperforms the NEWS in clinically relevant ED outcomes. The triage system demonstrated statistically significant superiority across all study endpoints, particularly for physician-assessed severity. This finding underscores that the NEWS, while useful for mortality prediction, is not a suitable replacement for triage systems.

Rather than seeking to replace triage systems, efforts should focus on identifying and addressing their weaknesses [5,6]. This was also emphasized by Zachariasse et al., who advocated refining existing triage models rather than replacing them [5].

An example of this approach is the South African Triage Scale, which integrated the NEWS to create the Triage Early Warning Score (TEWS) [6,20,21]. Instead of oversimplifying triage systems, this strategy enhanced patient stratification [20,21]. Similarly, efforts should be directed toward improving existing triage systems rather than replacing them with tools like the NEWS, which fail to capture crucial aspects of ED risk assessment. For instance, studies suggest that a three-level triage classification is insufficient, and a five-level system is necessary for optimal risk assessment [17].

Another critical issue is the fragmentation of research on triage systems. While dedicated working groups exist for optimizing the management of specific conditions such as chest pain, syncope, or sepsis, there is currently no coordinated international initiative for triage system optimization [22]. Instead, many studies propose modifications or additions tailored to specific patient subgroups, such as older or frail patients [7,9,10]. However, this approach risks making triage systems increasingly complex and fragmented, ultimately reducing their overall effectiveness [22].

Despite its imperfections, triage plays a fundamental role in assigning priority codes, particularly in overcrowded EDs with limited resources [16,17]. Therefore, rather than introducing fragmented and highly specific modifications, efforts should be directed toward substantial structural and functional improvements of triage systems.

Analyzing our descriptive and contingency tables reveals a notable discrepancy between the NEWS and triage-assigned severity. For example, among patients with NEWSs between 0 and 4, 7289 received a triage code indicating a severity level higher than 4. This discrepancy arises because, regardless of recorded vital signs, some patients present with severe symptoms requiring urgent evaluation [16]. A clear example is a patient with typical chest pain suggestive of acute myocardial infarction but without abnormal vital signs. Such a case cannot be considered low-risk, as emphasized by the European Society of Cardiology guidelines, which state that initial severity stratification should not rely solely on vital signs [23]. The same principle applies to patients with syncope or stroke, where clinical presentation is crucial for triage decisions [24,25].

Conversely, the NEWS may also overestimate the severity of patients with abnormal vital signs but no critical pathology. For example, a patient experiencing a panic attack may have an elevated heart and respiratory rate, resulting in a NEWS of 9, with the consequence of being categorized as high-risk, despite the absence of a life-threatening condition.

These findings highlight that relying solely on vital signs for triage stratification, as suggested by some recent studies, is inappropriate. A more effective approach involves integrating existing tools and progressively improving triage systems rather than attempting to replace them with early warning scores that fail to capture the complexity of initial patient assessment in the ED.

The present retrospective, single-center study has a few limitations, most related to its specific design. The decision to analyze 5% of patients from the overall population was made a priori, primarily to ensure the feasibility of detailed manual chart review by physicians and nurses. Given the large sample size, a complete case-by-case assessment of all patients would have been impractical. A 5% subset allowed for in-depth data extraction, including comorbidity scoring and clinical outcome reconstruction, while maintaining a representative sample of the overall cohort.

This approach is consistent with methodologies used in other ED-based studies that require detailed clinical assessments beyond routine electronic health record data.

## 5. Conclusions

This study demonstrates that the MTS outperformed the NEWS in classifying patient severity across multiple clinically relevant outcomes, except for 30-day mortality. While the NEWS is effective at identifying patients at risk of death, it lacks the specificity required for ED triage, as it does not account for symptomatology and clinical presentation. Given the low incidence of ED mortality, prioritizing the NEWS over traditional triage systems could lead to misclassification and inefficiencies in patient management. Rather than replacing the MTS or other triage systems with early warning scores, efforts should focus on integrating complementary tools to refine existing triage protocols.

## Figures and Tables

**Table 1:** Clinical and anamnestic characteristics of patients enrolled during the study period.

	NEWS 0–4	NEWS 5–6	NEWS ≥ 7	*p*-Value
Patients, *n* (%)	24.866 (91.3)	1.417 (5.2)	955 (3.5)	
Age in years, median (IQR)	59 (39–77)	74 (56–83)	79 (70–87)	<0.001
Arrival mode, *n* (%)				<0.001
Self-arrival	23,797 (95.7)	1210 (85.4)	757 (79.3)	
Ambulance	771 (3.1)	112 (7.9)	98 (10.3)	
Ambulance with physician	274 (1.1)	94 (6.6)	99 (10.4)	
Arrival time, *n* (%)				0.954
Day (08:00–20:00)	18.576 (74.7)	1.056 (74.5)	717 (75.1)	
Night (20:00–08:00)	6290 (25.3)	361 (25.5)	238 (24.9)	
Triage priority code, *n* (%)				<0.001
Priority 1	16 (0.1)	10 (0.7)	78 (8.2)	
Priority 2	1.519 (6.1)	451 (31.8)	563 (58.9)	
Priority 3	5.754 (23.1)	594 (41.9)	262 (27.4)	
Priority 4	15.604 (62.8)	337 (23.8)	51 (5.3)	
Priority 5	1.973 (7.9)	25 (1.7)	1 (0.1)	
Hospitalization, *n* (%)	3.986 (16.0)	684 (48.3)	711 (74.4)	<0.001
ICU admission, *n* (%)	398 (1.6)	56 (3.9)	44 (4.6)	<0.001
30-day mortality, *n* (%)	341 (1.4)	105 (7.4)	197 (20.6)	<0.001

**Table 2:** Predictive ability of the NEWS and the triage system evaluated using ROC curves for each study outcome in the general population.

	ROC	95% CI	*p*-Value
**30-day mortality**			<0.001
NEWS	0.745	0.726–0.764	
Triage	0.701	0.681–0.721	
**Hospitalization**			<0.001
NEWS	0.609	0.602–0.614	
Triage	0.733	0.725–0.740	
**ICU admission**			<0.001
NEWS	0.672	0.640–0.703	
Triage	0.862	0.840–0.882	

**Table 3:** Predictive ability of the NEWS and the triage system evaluated using ROC curves for each study outcome in the subgroup analysis.

	ROC	95% CI	*p*-Value
**Life-saving intervention**			<0.001
NEWS	0.751	0.639–0.864	
Triage	0.835	0.741–0.929	
**Clinical priority**			<0.001
NEWS	0.656	0.628–0.685	
Triage	0.857	0.832–0.880	
**Clinical severity**			<0.001
NEWS	0.661	0.629–0.692	
Triage	0.767	0.733–0.800	

## Data Availability

Data available on request due to privacy/ethical restrictions.

## Supplementary Materials

The following supporting information can be downloaded at: https://www.mdpi.com/article/10.3390/diagnostics15091055/s1, Table S1: Contingency table comparing triage codes with NEWS scores. NEWS is categorized into three severity levels, and the triage system has been grouped into three levels for direct comparison. White cells indicate where the NEWS score aligns with the triage score, while yellow cells denote a one-level discrepancy between triage and NEW. Orange cells highlight cases where the discrepancy exceeds one level.; Table S2: Clinical and anamnestic characteristics of the 5% patient subgroup selected for analysis.
