# Supplementary material for: Comparing the National Early Warning Score and the Manchester Triage System in Emergency Department Triage: A Multi-Outcome Performance Evaluation
Source: Diagnostics (Basel). 2025 Apr 22;15(9):1055. doi: 10.3390/diagnostics15091055 (PMC12071705; doi:10.3390/diagnostics15091055)
Supplement: Supplementary file 1 [file diagnostics-15-01055-s001.zip › diagnostics-3563558-supplementary.pdf]

	**NEWS 0-4**	**NEWS 5-6**	**NEWS ≥ 7**
**Triage priority 4-5**	17.577 (70.7)	362 (25.5)	52 (5.4)
**Triage priority 3**	5.754 (23.1)	594 (41.9)	262 (27.4)
**Triage priority 1-2**	1.535 (6.2)	461 (32.5)	641 (67.1)

Table S1: Contingency table comparing triage codes with NEWS scores. NEWS is categorized into three severity levels, and the triage system has been grouped into three levels for direct comparison. White cells indicate where the NEWS score aligns with the triage score, while yellow cells denote a one-level discrepancy between triage and NEW. Orange cells highlight cases where the discrepancy exceeds one level.

	**NEWS 0-4**	**NEWS 5-6**	**NEWS ≥ 7**	**p-value**
Patients, n (%)	1.231 (90.4)	71 (5.2)	60 (4.4)	
Age in years, median (IQR)	64 (43-79)	76 (64-87)	79 (68-84)	<0.001
Arrival mode, n (%)				<0.001
Self-arrival	1.184 (96.2)	61 (86.1)	47 (78.3)	
Ambulance	35 (2.8)	6 (8.4)	8 (12.6)	
Ambulance with physician	12 (1.0)	4 (5.5)	5 (9.1)	
Arrival time, n (%)				0.785
Day (08:00-20:00)	896 (72.8)	54 (75.4)	45 (74.2)	
Night (20:00-08:00)	335 (27.2)	17 (24.6)	15 (25.8)	
Triage priority code, n (%)				<0.001
Priority 1	2 (0.2)	1 (1.4)	3 (5.0)	
Priority 2	56 (4.6)	25 (35.2)	46 (76.7)	
Priority 3	292 (23.7)	31 (43.7)	5 (8.3)	
Priority 4	847 (68.8)	13 (18.3)	6 (10.0)	
Priority 5	34 (2.7)	1 (1.4)	0 (0.0)	
Charlson Comorbidity Index, median (IQR)	2 (0-4)	4 (3-6)	5 (4-7)	<0.001
APACHE, median (IQR)	1 (0-2)	3 (2-4)	7 (4-8)	<0.001
LSI, n (%)	9 (0.7)	2 (2.8)	10 (16.7)	<0.001
Clinical priority, n (%)	186 (15.1)	47 (66.2)	50 (83.3)	<0.001
Clinical severity, n (%)	147 (11.9)	39 (54.9)	44 (73.3)	<0.001

Table S2: Clinical and anamnestic characteristics of the 5% patient subgroup selected for analysis.
